# The Editor's Letter-Box

**Published:** 1914-01-31

**Authors:** 


*To the Editor of* The Hospital.

Sir,—I am not at all concerned to defend the new Poor-Law Orders, which, like all human efforts, are not free from blemishes, but the letter in your issue of the 24th instant, signed "A Workhouse Medical Officer," is about as foolish and unjustifiable a piece of "criticism" as I have seen for some time.

He entirely overlooks the fact that the classification of the ordinary inmates of the workhouse is largely based on "age, character, and behaviour," on which even a layman may be presumed to be as capable of judging as a workhouse medical officer; but, so far as the sick wards, the lunatic wards, and the nurseries are concerned, the medical officer reigns supreme, and has full and autocratic sway. His absurd references to Sub-division 4 of Article 10 are pretty clear evidence that he is determined to find an excuse for quarrelling with the Order, and is pretty hard put to it to find one. The power of the Guardians to which reference is made in the Article is a statutory power, and makes not the slightest alteration in the position of affairs which has existed on this point for nearly forty years. I can find nothing in the Order (neither can your correspondent) which will interfere with the exercise of his full and autocratic powers in dealing with the sick, which will apply to any inmate, whether married or single. As I said before, there is not the slightest change in the position which has existed for nearly forty years, and in an experience extending over twenty years I have never heard of any difficulty arising out of this power of the Guardians. Neither has your correspondent, I venture to say!

One is always glad to see an honest attempt to expose real grievances, but this captious criticism for the sake of criticising is neither good for the Poor-Law Service nor for the work it has to do.—Yours truly,

An Outdoor Poor-Law Officer.

